# Nature Studies in Macedonia

**Published:** 1917-08-18

**Authors:** 


### NATURE STUDIES IN MACEDONIA.

By AN OFFICER OF THE R.A.M.C. AND THE AUTHOR OF "NATURE NOTES."

Service in Macedonia has given me opportunity to observe land tortoises. Here they exist in such quantity that I often see a score or more in the course of a ride. As soon as hot days came, at the end of March, they emerged from their winter hiding-places. I know one kind only, but am told there are two. There is much variety of size and of colour and of marking of shell, but the number and arrangement of the pieces both of the upper and lower shell are constant. The under surface of the female is flat, or slightly convex outwards, of the male markedly concave outwards. This concavity appears to be a provision for convenience in mating. The male mounts the female for the purpose of impregnation in the fashion of four-footed mammals. The males are amorous and actively pursue the females, which always seem animated by desire to escape their attentions. When mating, the male utters a shrill, gasping squeak that is audible at some distance. As he cries he opens his mouth widely and jerks back his head in very quaint fashion. I have not discovered eggs, nor do I know how many a female may deposit nor where she deposits them. I occasionally find small tortoises about the size of a crown piece, but not many. I therefore suppose that the produce of each female is small, or else that there is great destruction of the very young by some enemy. When picked up, tortoises of either sex utter a low hiss of protest, and, the female only perhaps, exude often a quantity of a milky fluid from the anal region. The creatures feed chiefly on herbage. I have seen them grazing on several sorts of leaves and blades. I think they eat some kinds of lowly life—insects, grubs, snails, or suchlike—because I see them nosing about in places bare of grass or leaves. As they peer at you from the shelter of their shells they give an impression of intelligence and cunning. I suppose it is for this reason that in folk-lore tales the tortoise is seldom the fool. They are most plentiful in the foothills and the parts of the valley high enough to be dry. I never see any on the flat alluvial plain east of the river, and seldom on the similar soil on the western side. Tortoises hybernate in holes they dig for themselves in dry banks and in the sides of gullies. I suppose that any that stray into the low ground cannot survive the winter in water-logged hiding-places. The ground on either side the river is marshy, much of it even in summer, and most of it in winter.

They tend to accumulate in the bottoms of the gullies that cut through and through the surface of the foothills. This is, I think, because they slip into the gullies in their peregrinations and are not active enough to find a way out; since they love to be in the full blaze of the sun, the dark depths of many of the gullies scarcely can be congenial to them. They seem to like dry places and are as common far from water as near it, though they drink at times. I have seen one take a long drink from a little rill and afterwards crawl through it. In the marshes and stagnant ditches of the valley, the place of the land tortoise is taken by a species of water tortoise. This creature lies under the water all the winter, but in summer-time it seems to like to breathe the air, for I have often watched one poke its head above the surface as if to breathe, sink down, and appear in a short space, a minute or two, as if for another breath, and continue the motions for a considerable while. If these appearances are for the purpose of respiration, both expiration and inspiration would seem to take place above the surface, as I do not see bubbles rise from these reptiles such as rise from a diving mammal.

Yesterday, April 15, I saw a flock of bee-eaters. My attention was attracted to them by their rather plaintive callings. I found them flying at a considerable height in a small flock, hawking for insects. I saw many here last summer, but I had not observed them this year. They rest in holes in banks and in the walls of the gullies.

The kestrels and their graceful evolutions are a daily joy. As I rode through long grass, I put up a grasshopper of the size of a locust. It flew before my horse for some little distance. There was a kestrel hovering near by. It saw the grasshopper, slid swiftly from its place, and struck at it with its claws. Perhaps because it was keeping half an eye on me, the bird's stroke just failed. The grasshopper dropped like a stone to the security of the grass. The kestrel made no attempt to find it, but wheeled round and rose to and took its former position. At sunset as I returned I saw three kestrels near by that place hovering and dropping to seize something on the ground, rising, and transferring the prizes won from claw to beak by a graceful motion in the air. I got within a few yards of them, and time and again saw them drop, alight, grab with their claws, and rise. They sometimes came down within twenty or thirty feet of my horse. I am almost certain they were catching beetles. I thought I could see beetles in their claws. There is a kind of beetle about the size of a finger-nail that makes a hole to live in, into which it scuttles when alarmed outside. I think this was the food of the birds I was watching.

The human inhabitants of this country have curious customs in the disposal of their dead. The corpse is carried to the grave dressed in ordinary clothes, with the face uncovered. Bodies are buried in shallow graves and after a period dug up again, the bones being collected into a white linen bag and deposited in small buildings. One day I saw such a bag in a church; it was labelled with a woman's name. In a village near the Struma I have visited one of these store-houses of the bones of the departed. The bags most recently placed in it were still white and whole, others were whole but stained brown by time. Those that had been deposited in years past were rotted away and had spilled their contents, so there is a pile of the bones of many persons mixed in a gruesome heap, and on it rest the bags that contain the bones of the recently resurrected. I have inquired into the origin and meaning of these customs. I am told that when the Turks took Macedonia some five hundred years ago many persons escaped from persecution by causing it to be given out that they were dead, and evaded the Turks by a mock funeral. So the Turkish governors ordained that no bodies should be carried covered to the grave. The tyrannical ordinance became national custom, and as custom it has survived its first necessity. It is proposed to suppress the custom now it is recognised to be a relic of subjugation to Turkish rule.

I have found no satisfactory explanation of the other custom. It has been suggested to me that it is for convenience of assembly at the Last Day, but I do not consider that the statement was made with knowledge. I am told that when the bones are dug up they are examined, and, if flesh be found upon them, there is shame and tribulation in the family, for it is considered a sign that the dead has turned vampire and has retained flesh by sucking the blood of the living as they sleep. It is possible that the extreme anæmia that results from malaria may be the origin of belief in vampires. Nothing being known of the destruction of blood corpuscles by parasites and toxins, a conviction of a malignant and supernatural being draining the life-blood of the sufferer easily might have arisen, and even more easily have taken root amongst the ignorant and superstitious peasantry of the Balkan Peninsula.

